# Updates in Clinical and Genetics Aspects of Hypermobile Ehlers Danlos Syndrome

**DOI:** 10.4274/balkanmedj.2018.1113

**Published:** 2019-01-01

**Authors:** Irman Forghani

**Affiliations:** 1 Clinic of Clinical and Translational Genetics, Dr. John T. Macdonald Foundation Department of Human Genetics, University of Miami, Miller School of Medicine, Miami, USA

**Keywords:** Ehlers-Danlos syndrome, classification, joint hypermobility, hypermobility spectrum disorder

## Abstract

Efforts on recognition, diagnosis, and management of the presumed, most common connective tissue disorder hypermobile Ehlers-Danlos syndrome have been an ongoing challenge, even decades after the description of this condition. A recent international consortium proposed a revised Ehlers-Danlos syndrome classification, an update much needed since Villefranche nosology, in 1998. Hypermobile Ehlers-Danlos syndrome is the only subtype in these groups of syndromes with no known genetic cause(s). This effort brought significant attention to this often underappreciated condition. This review provides an update of the clinical and genetic aspects of hypermobile Ehlers-Danlos syndrome for clinicians and researchers.

Ehlers-Danlos syndromes (EDS) are a clinically and genetically heterogeneous group of heritable connective tissue disorders characterized by joint hypermobility (JH), skin hyperextensibility, and tissue fragility (1). EDS were first described by Hippocrates in 400 BC as a condition with joint laxity and multiple scars (2,3). In 1892, the Russian dermatologist Dr. Tschernogubow published the first comprehensive description of the syndrome (2,3). In 1901, the Danish dermatologist Edvard Ehlers and in 1908, the French dermatologist Henri-Alexandre Danlos each further defined the syndrome as a distinct entity. Eventually, in 1936, the English dermatologist Frederick Parkes-Weber suggested the name EDS (2,3). The first description of JH syndrome (JHS) was published in 1967 by Kirk et al. (4). Further classification of EDS into subgroups and delineation of hypermobile EDS (hEDS) are discussed below. The 2017 classification describes 13 subtypes of EDS, with hEDS being the most common subtype (1,5). hEDS is predominantly characterized by generalized JH (GJH), occurring within the first years of life, related musculoskeletal complications, mild skin hyperextensibility, mild aortic root dilatation, mitral valve prolapse, and easy bruising (5,6,7). Skin hyperextensibility in hEDS is milder than that in other forms of EDS such as classical EDS (cEDS) or classical-like EDS (clEDS). Other features associated with this syndrome include chronic pain, functional gastrointestinal disorders, Postural Orthostatic Tachycardia syndrome, and psychological dysfunction (1,5,6). These associated syndromes can be as debilitating as secondary complications of joint laxity (5,6). The diagnosis of hEDS is clinical and is established through a physical examination and a review of medical and family histories (1,5,6). The patients usually present with chronic pain, chronic fatigue, recurrent joint subluxations and dislocations, dysautonomia, functional gastrointestinal problems, and anxiety (5,6). A majority of the patients visit several specialists, seeking a diagnosis. hEDS behaves as an autosomal dominant syndrome (7). Some authors have reported a complete penetrance, whereas others observed incomplete penetrance and variable expressivity (5,6). One study on a large family proposed a 90% penetrance (8). The incidence of hEDS is higher among females than among males, and it is more commonly found in some populations such as Africans than in Caucasians (9,10). Castori and his group suggested that female predominance is related to differences in muscle pain perception related to the effects of sex hormones (9). Relevant to this topic, the effects of estrogen and estrogen-like compounds in the homeostasis of connective tissue and the knowledge gap in this area have been recently proposed, thereby opening an interesting area of research (11,12).

## Ehlers-Danlos Syndrome Classification

The first classification of EDS was established in the 1960s (3). In 1988, Beighton et al. (13) published a revised classification known as the “Berlin nosology,” which was proposed at the seventh International Congress of Human Genetics in Berlin in 1986. In the Berlin nosology, 11 EDS subtypes were defined, primarily based on the mode of inheritance and clinical presentations. They designated a Roman numeral to each subtype (14). In 1997, with further elucidation of the molecular causes of each EDS subtype, the Villefranche nosology stemmed from a meeting of experts in this field at Villefranche-Sur-Mer in France. The Villefranche nosology was published by Beighton et al. (14) in 1998. This nosology delineated six major subtypes for EDS and described the major and minor criteria for each subtype. Recently, an independent group of experts in this field created the international EDS consortium, and their efforts resulted in the latest classification of EDS in 2017 (1). Their proposed nosology is based on characteristic clinical manifestations and molecular and causative genetic variants in each EDS subtype; the sole exception is the hEDS subtype, the molecular cause of which remains unknown. The consortium defined 13 EDS subtypes, which are caused by pathogenic variants in 19 different genes, and revised the diagnostic criteria for hEDS (1,15). They proposed their nosology using a new nomenclature, emphasizing the description of each subtype, and designated a descriptive name and acronym to each subtype. Therefore, what was previously known as Ehlers-Danlos hypermobility or type III EDS is now identified as hypermobile EDS or hEDS.

## Diagnosis of Hypermobile Ehlers-Danlos Syndrome Based on the 2017 Diagnostic Criteria

Despite the significant advancements in molecular genetics, efforts toward the discovery of the genetic cause(s) of hEDS have yielded inconclusive results. The lack of success in identifying the molecular etiology of this condition is due to the heterogeneity and the variable expressivity of the condition, as well as the nonspecific phenotypes that overlap with other heritable connective tissue disorder and non-heritable disorders of connective tissue (1). The Villefranche nosology did not clearly define the required inclusive and exclusive criteria for establishing the diagnosis of hEDS. The criteria were divided into major and minor, with the major criteria including skin hyperextensibility and GJH and the minor criteria being chronic pain, recurrent joint dislocation, and positive family history. GJH refers to hypermobility of at least five or more joints, at the same time, usually at four limbs and the spine (16,17). The 2017 diagnostic criteria for hEDS are more specific and exclusive. A useful checklist of diagnostic criteria for hEDS is available on the EDS society website (https://ehlers-danlos.com/wp-content/uploads/hEDS-Dx-Criteria-checklist-1.pdf). The DazzleVegas 2017 Global Learning Conference videos presented by the EDS society provide detailed discussions about the new diagnostic criteria and their proper implementation (https://www.ehlers-danlos.com/2017-eds-global-conference/). Fulfillment of the following three criteria concurrently is required for establishing the hEDS diagnosis (1):

-  Criterion 1 is the presence of GJH.

-  Criterion 2 consists of three separate features (A, B, and C); feature A is systemic manifestations related to heritable connective tissue disorder, feature B is a positive family history, and feature C includes pain and secondary musculoskeletal complications of joint laxity.

-  Criterion 3 is for the exclusion of other heritable and acquired causes of hypermobility and possible alternative diagnoses that can present with JH.

Criterion 1: The Beighton scoring system, which was established in 1973, continues to be the most reliable assessment tool for GJH (16,17). A Beighton score ranges from 0 to 9 (Table 1) and is influenced by age, sex, ethnicity, history of trauma, and physical fitness of the person (16,17). Furthermore, for conditions that affect the Beighton score, such as previous surgeries and amputation of the joint, a self-reported five-point questionnaire [(5PQ), Table 2] is used to consider historic hypermobility (18,19). The consortium defined an age-adjusted cut-off value to consider the age-related changes in GJH. A cut-off point of ≥4 for men and women aged >50 years, a cut-off point of ≥5 for adults aged till 50 years, and a cut-off point of ≥6 for prepubertal children and adolescents confirm the presence of GJH. In addition, to take the historic JH into account, the committee decided to add one point to the Beighton score, when it is one point below the cut-off point and if two or more items in the 5PQ are positive (1). Experts recommend using an orthopedic goniometer to increase the accuracy of the GJH assessment (https://www.ehlers-danlos.com/assessing-joint-hypermobility/). Criterion 2 (features A-C). At least two of the features must be positive in the patient to meet criterion 2 (1).

-  Feature A (Table 3) requires at least five positive items.

-  Feature B is a positive family history (one or more first-degree family members who meet the 2017 diagnostic criteria for hEDS; therefore, the reported family history requires further investigation).

-   Feature C at least one must be positive, including daily musculoskeletal pain in limb(s), for 3 or more months, and/or chronic generalized pain for 3 or more months, and/or recurrent joint dislocation or frank joint instability not caused by trauma. It should be noted that feature C cannot be counted for those patients who have been previously diagnosed with an autoimmune disorder; instead, they must fulfill features A and B. Criterion 3 is aimed at the exclusion of other connective tissue disorders. All the following three prerequisites must be met: the absence of unusual skin fragility (consider other forms of EDS in the presence of unusual skin fragility), the exclusion of other heritable and acquired (autoimmune disorders) connective tissue disorders, and the exclusion of other heritable causes of JH. Molecular testing, which is not recommended for the diagnosis of hEDS, can be considered for the exclusion of other heritable causes of JH if there are any clinical concerns (1).

## Hypermobility Spectrum Disorder

In 1999, Grahame proposed that benign or asymptomatic JH and JHS are possibly the two ends of one spectrum (20). Furthermore, several experts have suggested that JHS and hEDS, which were initially introduced as two separate clinical entities, were actually equivalent conditions (21,22). Recently, Castori of experts elegantly described a dynamic framework for the classification of JH and the genetic syndromes featuring JH without a known molecular etiology. They merged these conditions into one umbrella of diagnosis and introduced the new terminology of hypermobility spectrum disorders that fills the gap between asymptomatic JH and hEDS. Hypermobility spectrum disorder includes all the conditions featuring JH plus one or more of its secondary musculoskeletal manifestations and is a diagnosis of exclusion for patients with symptomatic JH who do not meet the diagnostic criteria for other types of EDS or hEDS (23). It was indisputable that embodiment of a more selective diagnostic criteria for hEDS would exclude many of the individuals with symptomatic JH. Hypermobility spectrum disorder is an alternative diagnosis for this group of patients.

## Genetic Research on Hypermobile Ehlers-Danlos Syndrome

Creating a more homogeneous cohort for scientific research was one of the primary goals of the consortium in refining the diagnostic criteria for hEDS (1,23). Previous and recent studies favor the clinical and genetic heterogeneity of this syndrome, making the identification of its genetic etiology more difficult (24,25). A chronological review of the relevant studies is presented below. In 1994, Narcisi et al. (26) reported the first mutation in five affected members of a family with clinical characteristics of hEDS who carried a pathogenic variant in the *COL3A1* gene. None of them had history of vascular fragility, characteristic facial features, or any other characteristics of vascular EDS (vEDS). This *COL3A1* variant has not been reported in any other cases of hEDS and is noted in the ClinVar database as EDS, Nonvascular variant [NM_000090.3(*COL3A1*):c.2410G>A (p.Gly804Ser)] (https://www.ncbi.nlm.nih.gov/clinvar/variation/17221/). A 2004 twin study on 483 monozygotic and 472 dizygotic female twin pairs, conducted in the UK, reported an estimated heritability of 70% and a higher concordance for JH in the monozygotic twins (60%) than in the dizygotic twins (36%), suggesting that JH has a highly significant genetic component (27). In 2001, Schalkwijk et al. (28) introduced a subtype of EDS that is now known as clEDS. clEDS is an autosomal recessive form of EDS and is caused by a deficiency of tenascin-XB, an extracellular matrix glycoprotein encoded by the *TNXB* gene. The same group later described an association between haploinsufficiency of the *TNXB* gene and JH in heterozygous females. They evaluated 20 heterozygous family members comprising 14 females and 6 males. All the subjects showed a significant decrease in serum tenascin levels, although 9 of them, all females, showed GJH, when evaluated using the Beighton scoring system. Despite this reduction in serum tenascin levels, none of the heterozygous males presented with JH (29). In 2013, Merke et al. (30) described a contiguous gene deletion syndrome caused by deletion of *CYP21A2* and its flanking gene *TNXB*, and they termed it as congenital adrenal hyperplasia-X syndrome. Congenital adrenal hyperplasia-X syndrome presents with combined phenotypes of congenital adrenal hyperplasia and hypermobility. The authors associated the hypermobility phenotype of this syndrome to haploinsufficiency of the *TNXB* gene (30). In 2015, Morissette et al. (31) found a heterozygous missense variant, c.12174C>G (p.C4058W), in the *TNXB* gene in 10 people from seven families with congenital adrenal hyperplasia-X. They showed that this variant does not affect the protein expression of tenascin in dermal fibroblasts and proposed a dominant-negative mechanism for this missense variant, which is different from the haploinsufficiency caused by deletion or truncated mutations previously reported. The molecular analysis of the *TNXB* gene is challenging due to the presence of a pseudogene. Most of the available gene panels do not include this gene, which may impact the real estimate of the frequency of this gene in patients with hEDS. In 2015, Syx et al. (8) performed the first genome-wide linkage analysis in a large Belgium family with hEDS and suggested an 8.8-Mb candidate linkage interval on chromosome 8 (8p22-8p21.1), with a maximum two-point LOD score of 4.73. Whole exome sequencing of two affected family members subsequently identified a missense variant in the leucine zipper, putative tumor suppressor-1 gene (*LZTS1*) in this region (p.His211Gln). All the affected, and none of the unaffected, individuals in this family harbored the same variant in *LZTS1*. However, the authors did not find the 8p22-8p21.1 locus linkage in four other hEDS families. Interestingly, sequencing of the *LZTS1* gene in 230 additional unrelated individuals with hEDS identified three additional variants in this gene (8). They concluded that *LZTS1* variants are associated with a small percentage of patients with hEDS (2% in their cohort), thereby confirming the genetic heterogeneity of this disorder.

In 2016, at the University of Brescia, Italy, Colombi and her group conducted an *in vitro* comprehensive immunofluorescence analysis and transcriptome-wide expression profiling using cultured fibroblasts of five affected females with JHS and hEDS (32). Their study demonstrated disorganization of collagens and fibronectin and their integrin receptors, along with a widespread disarray of several matrix structural components (32). Furthermore, they demonstrated altered expression of multiple genes involved in homeostasis signaling cascades, maintenance of extracellular matrix architecture, cell-cell adhesion, and inflammatory/immune/pain responses. This observation in hEDS/hypermobility spectrum disorder was consistent with their previous findings in cEDS, vEDS, and the majority of other EDS types (33). In addition, they reported overexpression of an alternative fibronectin (avβ3 integrin) receptor in the fibroblasts of patients with hEDS or JHS similar to that in patients with cEDS and vEDS (32,33). They had previously proposed the compensatory role of the avβ3 integrin receptor in anoikis (apoptosis induced by extracellular matrix disassembly) rescue in *COL5A1* and *COL3A1* mutations associated with cEDS and vEDS, respectively (33). These studies agree with previous works describing the clinical and genetic heterogeneity of this syndrome (8). The same group recently published a follow-up study regarding cellular characterization of dermal fibroblasts in patients with hEDS or JHS (34). They described an exclusive myofibroblast-like phenotype in *in vitro* dermal fibroblasts of patients with hEDS/hypermobility spectrum disorder, in contrast to other types of EDS, and the role of the avβ3 integrin receptor in this phenotype, in addition to the anoikis rescue. They also hypothesized that a constellation of factors such as persistent expression of the alpha-smooth muscle actin cytoskeleton, as well as high levels of the active form of matrix metallopeptidase-9, along with the role of inflammatory mediators and altered expression of N-cadherin family could be involved in the pathogenesis of hEDS/hypermobility spectrum disorder and the formation of the myofibroblast-like phenotype (32,34). They suggested that matrix metallopeptidase-9, which is involved in the physiological degradation of the extracellular matrix, plays a role in fibronectin fragmentation and extracellular matrix disassembly in patients with hEDS/hypermobility spectrum disorder (34). Alpha-smooth muscle actin cytoskeleton is expressed during wound healing and tissue regeneration and is required for the physiological transition of fibroblasts to myofibroblasts (35). Furthermore, Colombi and her group proposed a regulatory effect of cadherin on the expression of alpha-smooth muscle actin cytoskeleton through the Wnt/b-catenin pathway, the involvement of avβ3 integrin-ILK-mediated signal transduction and Snail/Slug in the overexpression of matrix metallopeptidase-9, disorganization of alpha-smooth muscle actin cytoskeleton and fibroblast to myofibroblast transition, and subsequently, the pathogenesis of hEDS or hypermobility spectrum disorder. Interestingly, they observed the myofibroblast-like phenotype in control cells grown in conditioned media of hEDS/hypermobility spectrum disorder cultures, suggesting the proteolytic activity of conditioned media in the myofibroblast-like phenotype, similar to a chronic inflammatory state. Therefore, they suggested that this multisystem disorder is a part of a sequence of altered extracellular matrix and chronic inflammatory state, whereas a a distinct clinical entity (34). In 2018, Colombi and her group summarized their work about the key role of avβ3 integrin and its different role through specific signaling pathways in each EDS subtype based on their molecular basis (36). However, a better understanding of these complex pathways is needed to elucidate the pathogenesis of these conditions.

## Future Directions

The exact prevalence of hEDS is not clear (37). Application of the recent diagnostic criteria and clear exclusion of hEDS from hypermobility spectrum disorder entail future observational studies for achievement of an accurate prevalence of these conditions.

Determination of GJH in the new diagnostic criteria for hEDS is adjusted according to the age. However, the implication of age into other recently introduced criteria is not clear. Some phenotypic features of hereditary connective tissue disorders emerge as age progresses. Longitudinal studies are needed for better understanding of the temporal nature of hEDS and hypermobility spectrum disorder. Recent studies on cultured fibroblasts from a small number of adult females with hEDS were suggestive of an inflammatory-like condition and “a phenotypic continuum rather than a distinct clinical entity” (32). Further studies on younger individuals diagnosed with hEDS or hypermobility spectrum disorder would help in distinguishing between secondary vs primary inflammatory state. The prevalence of JHS is estimated to be around 2%, and a twin study suggested a heritability of 70% (27,37). Similarly, the prevalence and heritability of schizophrenia are 1% and 80%, respectively (38,39). Genome-wide association studies in schizophrenia were successful in identifying multiple gene loci and candidate genes (40,41). A multicentric genome-wide association study on hypermobility spectrum disorder and hEDS could identify novel candidate loci in this spectrum of disorders.

## Figures and Tables

**Table 1:**
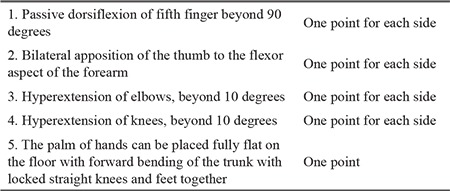
Beighton scoring system (14)

**Table 2:**
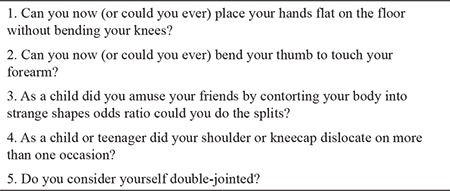
5-point questionnaire (18)

**Table 3:**
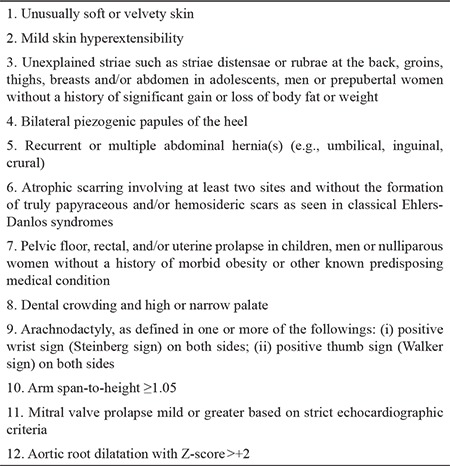
Feature A, Criterion 2, the 2017 diagnostic criteria for hypermobile Ehlers-Danlos syndromes (1)
